# Species specific exome probes reveal new insights in positively selected genes in nonhuman primates

**DOI:** 10.1038/srep33876

**Published:** 2016-09-23

**Authors:** Zheng Su, Junjie Zhang, Chanchal Kumar, Cliona Molony, Hongchao Lu, Ronghua Chen, David J. Stone, Fei Ling, Xiao Liu

**Affiliations:** 1 BGI-Shenzhen, Shenzhen, Guangdong 518083, China; 2 Shool of bioscience & bioengineering, South China University of Technology, Guangzhou, Guangdong 510006, China; 3 Translational Medicine Research Centre, Merck Research Laboratories, MSD, 8 Biomedical Grove, Neuros #04-01, Singapore 138665, Singapore; 4 Merck Research Laboratories, Merck & Co. Inc., 33 Avenue Louis Pasteur, Boston, MA 02115, USA; 5 Informatics IT, MSD R&D (China) Co., Ltd., Beijing, China; 6 Informatics IT, Merck & Co., Inc., Boston, MA, USA; 7 Merck Research Laboratories, Merck & Co. Inc., 770 Sumneytown Pike, WP53B-120 West Point, PA 19486, USA; 8 Department of Biology, University of Copenhagen, Copenhagen 2200, Denmark

## Abstract

Nonhuman primates (NHP) are important biomedical animal models for the study of human disease. Of these, the most widely used models in biomedical research currently are from the genus *Macaca*. However, evolutionary genetic divergence between human and NHP species makes human-based probes inefficient for the capture of genomic regions of NHP for sequencing and study. Here we introduce a new method to resequence the exome of NHP species by a designed capture approach specifically targeted to the NHP, and demonstrate its superior performance on four NHP species or subspecies. Detailed investigation on biomedically relevant genes demonstrated superior capture by the new approach. We identified 28 genes that appeared to be pseudogenized and inactivated in macaque. Finally, we identified 187 genes showing strong evidence for positive selection across all branches of the primate phylogeny including many novel findings.

Nonhuman primates (NHP), including chimpanzee and old world monkeys such as rhesus and cynomolgus macaque, are important biomedical animal models for the study of human evolution and diseases due to their close genetic background. Rhesus is the primary animal model used in translational and biomedical research in pharmacology, neuroscience, infectious disease, immunology, tissue engineering, gene therapy, senescence and learning^1^. Nonhuman primate disease models have played a critical role in elucidating the etiology, mechanism, treatment and progression of various human disease^2^,^3^,^4^,^5^. The first Indian rhesus genome was sequenced and assembled in 2007^6^, providing a genomic reference map for the study of rhesus inheritance and diversity. Human genomic diversity has been explored by the HapMap and 1000 Genome Project after the first human genome was sequenced. For the nonhuman primates, the lack of available tools has hampered the progress of genome-wide, large-scale studies to uncover genetic diversity.

Human exome sequencing^7^,^8^,is widely used to study population polymorphisms, genetic inheritance in Mendelian disorders and complex diseases. In light of the need to study the NHP exome, several recent studies have attempted to adopt human exome based approaches directly^9^,^10^,^11^,^12^, two of which had been successfully applied to study adaptive selection in primates^9^,^11^. However, genetic divergence between humans and NHP may result in reduced efficiency and accuracy when studying protein coding regions in NHP via human based capture and sequencing. Across the genome, an estimated 6.46% divergence exists between humans and rhesus, and this is increased to 9.24% when taking into account small insertions and deletions^6^. At the gene level, up to 4% divergence can be displayed between the two species^9^. Consequently, the hybridization kinetics for enrichment will be lower as a result of the mismatches between the target and probe sequences, especially for those highly divergent genes, such as those under strong selection in either lineage.

Adaptive evolution of protein coding genes in primates sheds light on substantially important biological questions including population diversity, migration and predisposition of complex traits. The identification of genetic variation in protein coding regions by high throughput exome sequencing gives rise to an unprecedented opportunity to study the genetics of adaptation, in particular to analyze genes under positive selection. A genome-wide scan of the first assembled rhesus genome, in comparison with human and chimpanzee coding sequences, identified a union set of 178 genes under positive selection on at least one of these lineages^6^. Exome sequencing on Old World and New World monkeys revealed new targets of positive selection^9^ and a study on the chimpanzee uncovered the extensive adaptive selection linked with X chromosome^11^. Notably, a similar human exome-based approach was adopted in the latter two studies, which not only revealed the importance and usefulness of the approach but also highlighted the previously discussed drawbacks, including a bias towards filtering fast-evolving genes, and an underestimation of the prevalence of positive selection.

To address these limitations, we have designed a set of probes that directly target the annotated protein coding regions in the rhesus and cynomolgus macaque genomes, and then applied it to enrich and sequence the exomes of four species or subspecies of NHP, including Chinese rhesus macaque (*Macaca mulatta lasiota,* CR), cynomolgus macaque (*Macaca fascicularis*) of Vietnam origin (CC), Indonesian cynomolgus macaque (IC) and Mauritian cynomolgus macaque (MC). By comparing our macaque specific exome probes with the human exome capture approach on the same samples, we have demonstrated superior performance of our methodology in reproducibility, efficiency, gene coverage, sensitivity and accuracy in identifying variants. As a result, we have identified 28 genes putatively pseudogenized in macaque, which cluster in biological categories of olfactory sensory and G protein coupled receptor (GPCR) signaling. Finally, from the comprehensive exome data of the four above macaque subspecies, in combination with other NHP species, we did a whole exome scan for genes under positive selection at any time of primate evolution, and discovered new sets of genes, which demonstrated the usefulness of the new approach.

## Results

### Characterization and merging of the exome sequences for rhesus and cynomolgus macaque to design probes

The species primarily targeted by our new exome capture approach are from the genus *Macaca*, which include the most widely used NHPs in biomedical research. In order to design the optimal set of probes, we selected two representative species with available whole genome data and high-quality protein coding annotations: the Chinese rhesus macaque (*Macaca mulatta lasiota*) and cynomolgus macaque (*Macaca fascicularis*)^13^. Genomic sequences and annotations from cynomolgus macaque were collected, including 21,283 genes with 187,619 coding sequences in the total length of 31.2 Mb, while Chinese rhesus genome contains 21,610 genes with 189,751 coding sequences in the length of 32.0 Mb. After merging highly similar target regions, 35.9 Mb sequences were generated for probe design. With flanking 100 bp on upstream and downstream of reference, probes for 52.1 Mb target region with 94.4% theoretical sequence coverage were generated by Roche Nimblegen according to their custom design protocols.

### The new approach provides better uniformity, sequence coverage on exome and biomedical genes

**Figure 1:**
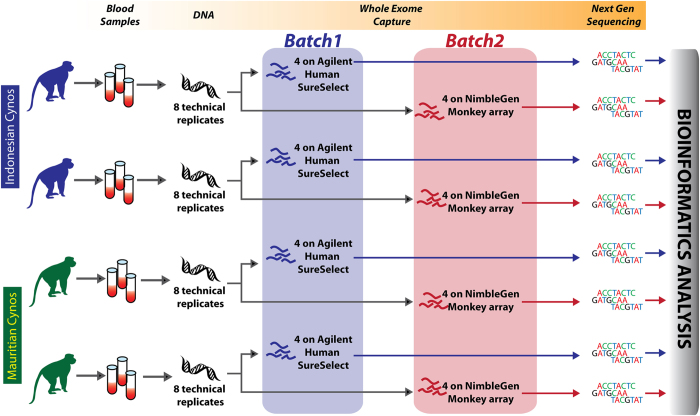
The experimental design for methods comparison.

We designed and performed a systemic experiment to comprehensively compare and evaluate our new approach (Monkey Probes, MP) with the human exome probes based methodology (Human Probes, HP, details in Methods). Two ICs and two MCs (four primates in total) were selected for exome sequencing by both MP and HP pipelines, with four technical replicates per sample (Fig. 1). All samples were sequenced to a coverage depth that exceeded 100x (Table S1). In addition, two CRs and two CCs were sequenced to 90–100x with MP for the positive selection analysis (Table S1). To exclude the influence of depth, we randomly extracted the same amount of data for all samples for analysis. We first evaluated the reproducibility of both pipelines by correlation analysis between replicates based on the sequence coverage of each gene, and found that both approaches showed high reproducibility, while MP was marginally better (Pearson correlation coefficient r = 0.997 ± 0.001 vs. 0.994 ± 0.001, Figure S1).

Read distribution is critical for efficient exome enrichment and sequencing, since for any given amount of sequencing data, increased uniformity of coverage depth results in more efficient variant calling across a wider range of exons. For this reason, we next compared uniformity of reads distribution between MP and HP. In order to exclude the impact of assembly quality from the reference genome, for ICs and MCs, genomes of Indian rhesus (IR) and matched subspecies of Cynomolgus (CE) were used for alignment (Table 1). In both IR and CE alignments, the single nucleotide depth distribution of MP showed a curve closer to Gaussian distribution than did HP (Figure S2), which indicated less variability in capture efficiency across all the target regions for MP relative to HP. We then took a deeper look at each chromosome, and demonstrated that in both IR and CE alignments MP reads were more evenly distributed across all chromosomes, with the mean and median depth for each chromosome almost unanimously higher in MP than HP when sequenced with same amount of data (Figure S3).

**Figure 2:**
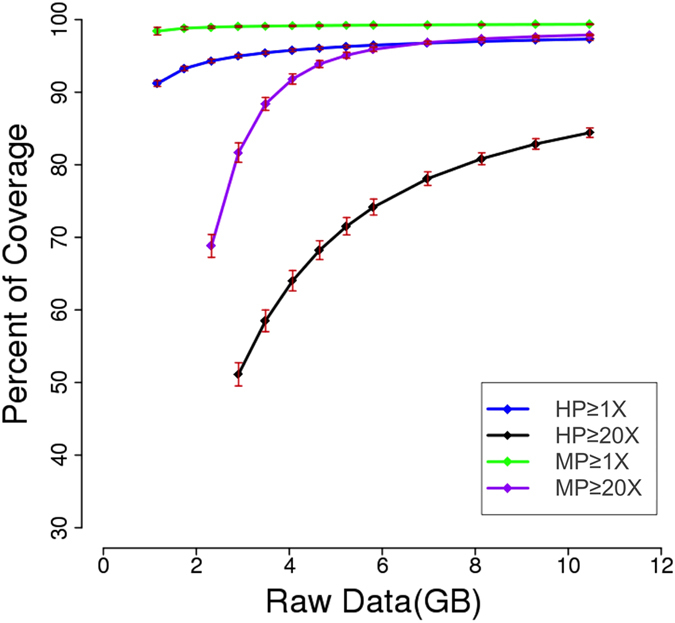
The exome coverage of Human probes (HP) and Monkey Probes (MP) in correlation with sequence data amount. Shown in the figure is the percentage of whole exome covered by one read (1x) and twenty reads (20x) in HP and MP platform, by reads aligned to cynomolgus genome. With same amount of raw sequence data, the 20x exome coverage is significantly higher in MP than HP.

We hypothesized our new exome sequencing would provide better sequence coverage, especially for genes with high measurements of divergence between human and NHP. When looking at the overall 1x read coverage on whole CE exome, MP and HP were comparable (Table S1). The read coverage at 1x gives an estimate of the percentage of the target region which can be captured, while only read coverage above a certain threshold is meaningful for high-confidence variation calling. At coverage depths ≥20x, the power of the new approach is apparent. We plotted a saturation curve (Fig. 2) which revealed the association between the amount of raw data and the percentage of exome region covered with ≥1 read and ≥20 reads. From the curve, it is clear that even with substantial raw data (11.63 Gb), HP can only cover 80–90% of the exome with ≥20 reads. However, MP was able to capture almost 90% of exome with ≥20 reads from as low as 4 Gb raw data, and reached saturation at 8 to 10 Gb with 95% exome being covered with ≥20 reads. Exome coverage with ≥20 reads was substantially higher in MP than in HP with the same amount of data, indicating MP was more efficient in calling exome-wide variants.

**Figure 3:**
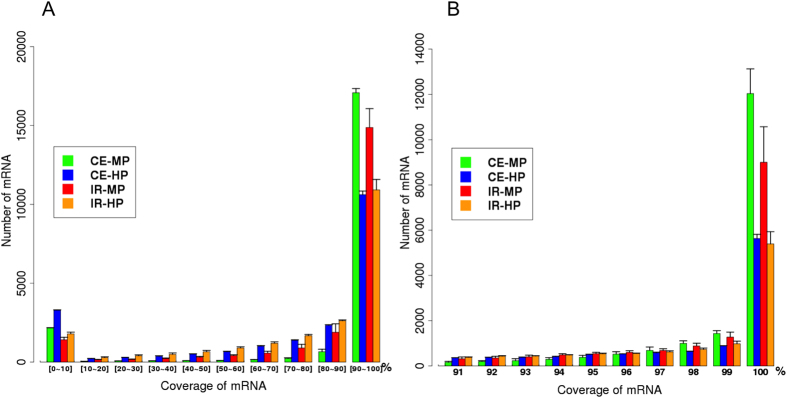
The distribution of coverage in genes by at least one read. Bars of different color correspond to performance of MP and HP platform with reads aligned to Indian rhesus reference genome (IR) and cynomolgus genome (CE). For example, green bar (CE-MP) indicates the MP platform with reads aligned to cynomolgus genome. The height of each bar represents the number of genes with the corresponding coverage. (**A**) distribution of genes with coverage from 0–100%, divided by 10 windows of 10%. (**B**) distribution of genes with coverage ranging from 90–100%. In both CE and IR alignment, MP tends to provide a more complete gene coverage than HP.

The comparison of uniformity and coverage illustrated that in some exonic regions or genes MP enabled more efficient capture than HP. We then did a more thorough evaluation of these genes. For the 20,950 genes in IR genome and 21,283 genes in CE genome, the proportion of transcribed gene region aligned by at least twenty reads (defined here as gene coverage) was evaluated and compared between MP and HP. Not surprisingly, more genes in HP were only aligned at 0–10%, while MP platform ensured more genes to be captured at 90–100% (Fig. 3a). Even for the latter genes, MP revealed a clear trend towards more complete gene coverage when compared with HP. Additionally, MP had greater number of genes achieving 100% gene coverage, both in IR and CE alignment (Fig. 3b). Under the definition of a failed gene as having less than 20% gene coverage and a successful gene as having greater than 80% gene coverage, 500 genes were successful in MP but failed in HP, as opposed to 223 genes in the reverse comparison, in IR alignment. All these genes are listed in Tables S2 and S3.

**Figure 4:**
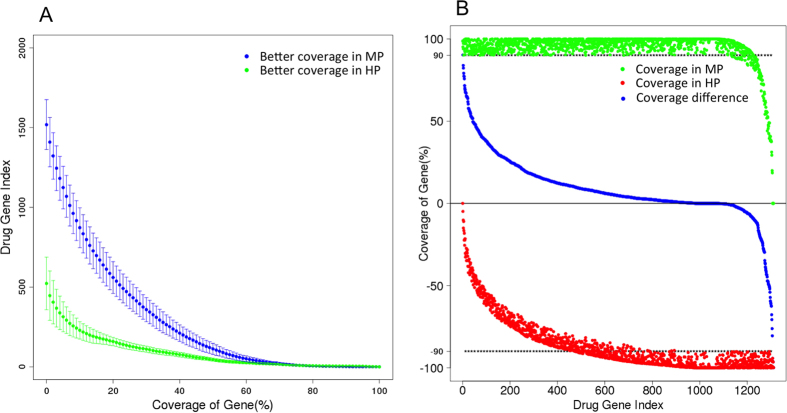
Coverage of biomedically relevant genes. (**A**) x-axis is the percentage difference of coverage on biomedical genes between MP and HP, while Y-axis is the cumulative number of genes with the corresponding difference of gene coverage. Blue curve illustrates the distribution of genes with better coverage in MP, while green curve illustrates the genes with better coverage in HP. Mean value and Standard Deviation are showed from all the replicates of all the samples. (**B**) Gene coverage in MP and HP platform for sample MC1-1. Shown are all the biomedical genes covered above 90% by at least one read in MP or HP platform. Green dots are the coverage of each gene in MP, and red dots are the coverage in HP. Blue curve corresponds to the coverage difference of each gene.

Next, we examined biomedically relevant genes, to ascertain whether MP performed better on this subset. Genes labeled as transporters, carriers, enzymes and drug targets were downloaded from Drugbank database^14^, and 2,146 genes were found to have orthologs in macaque genome (details in Methods). We investigated the sequence coverage of these genes for all the samples in MP and HP by aligning to the human genome. More genes were found to have better gene coverage in MP than HP (Fig. 4A). For all the 2,146 biomedical genes, approximately 75% were better in MP. For example, with sample MC1-1, we found 985 genes with ≥90% gene coverage using MP, but only 240 genes with ≥90% gene coverage using HP (Fig. 4B and Table S4). Therefore, MP provided superior performance by ensuring better sequence coverage for a majority of selected genes of interest in biomedical research.

### Psuedogenized genes or putative dysfunctional genes in macaque

With the increased power of our new approach, we identified genomic variants in all of our samples, including SNPs and short indels (1–15 bp). The numbers of exonic SNPs and indels called are listed in Table S5, based on alignments on the IR genome assembly. On average, 75,445, 65,220, 89,208, 84,920 SNPs and 1,941, 1,779, 2,057, 1,956 short indels per sample were called from CC, CR, IC and MC, respectively. We analyzed the length of coding indels called from the IR genome reference, and found the length distribution of coding indels had an enrichment of 3N (N = 1, 2, 3…), in which reading frame was preserved (Figure S4). This was consistent with the previous report for NHP^9^, and suggested the accuracy of our indel calling. In order to test the quality of our SNP calling, sixteen SNPs were randomly selected for PCR and Sanger sequencing validation, and all of these (16/16) were validated (Table S6).

Next, we compared the macaque genome with the human genome. For all exome-sequenced macaques, we identified 25 single-copy genes represented in the human genome, which contain homozygous SNPs in the macaque exomes. Since these particular SNPs introduce premature stop-codons or frameshift indels (Table S7 and details in Methods), this finding suggests that at least a subset of these genes were pseudogenized and inactivated in macaque populations (or conversely, that human populations experienced gain of function mutations) over the course of primate evolution. Functional annotation revealed that these genes represent several important categories including olfactory sensation and *GPCR* signaling. Of note, attention should be paid to several genes when macaque is adopted as the animal model to study human disease. *PSORS1C1*, which contained a homozygous stop-codon in all our macaque samples, was predicted to be pseudogenized. It was further validated by PCR plus Sanger sequencing on additional samples (Table S7). *PSORS1C1* is one of the several genes which confer susceptibility to psoriasis^15^ and systemic sclerosis^16^ by linkage and genome-wide association analysis in human. A premature stop-codon in *PSORS1C1* is found in gorilla genome^17^, but is not reported in the rhesus and cynomolgus genomes. Another example is *CD1E* encoding a T-cell surface glycoprotein, which in soluble state binds diacetylated lipids and is required for the presentation of the glycolipid antigens. *CD1E* contains a premature stop-codon which is validated by PCR (Table S7).

### Positive selection analysis

The superior performance of our new approach, together with the existing coding sequences of other primates, compelled us to further study any genes which showed strong signal of positive selection in primates. We obtained a set of 13,503 orthologous gene sequences, which contained no nonsense or frameshift mutations, from at least six species and subspecies including human, chimpanzee, mouse, rat, IR, CR, CC, IC and MC. In order to detect genes undergoing positive selection across all primate lineages, we used a primate phylogeny consisting of human, chimpanzee, IR, CR, CC, IC and MC. We then employed the M7-M8 model comparison using codeml of the PAML software package, which performs Phylogenetic Analysis by Maximum Likelihood^18^ (details in methods).

At false discovery rate (FDR) < 0.1, we found evidence of positive selection for 187 genes (Table S8, details in Methods), of which the top 50 are listed in Table 2. We tested if any chromosome showed an excess of genes under positive selection, especially the X chromosome^19^, but didn’t identify any chromosomes enriched (Table S9). To examine the consistency of our result with the previous findings, we compared the 67 positively selected genes across all branches of the phylogeny (FDR < 0.1) identified from the work of Gibbs *et al*.^6^ with our results. The additional cynomolgus branch included in our study on top of their phylogeny (human, chimpanzee and rhesus macaque) should increase our power to detect positive selection. Among the 67 genes, 44 genes were qualified in our ortholog dataset, and these genes ranked significantly higher in our scan for positive selection (P < 2.2 × 10^−16^, two sided Mann-Whitney U test). Four genes showed strong evidence for positive selection with an FDR < 0.1, and another 27 genes were also significant (P < 0.05). Overall, 31 out of 44 genes (70%) showed evidence for positive selection in our analysis. We also compared our results with those from George *et al*.^9^, which used human-based exome sequencing with a larger number of other species in the phylogeny. Among the 157 genes they identified as being positively adaptive across all branches (FDR < 0.1), 109 genes were also found in our orthologs, and 44 genes showed evidence for positive selection in our analysis (P < 0.05). Although we may have lost some power to detect positive selection due to our relatively narrow phylogeny, we were still able to access abundant novel genes as being adaptive. To examine whether this was due to the power of the species specific approach, we compared the sequence divergence between human and NHP for the positively selected genes identified in our analysis to the gene set from George *et al*.^9^, and found that our genes were significantly more divergent (P = 0.0032, Wilcoxon rank sum test; maximal divergences among the species tested). The genes specifically identified by us were also significantly more divergent (P = 0.0034, Wilcoxon rank sum test), implying the capability of the new approach to capture the rapidly evolving genes.

Genes related to cell adhesion and signal transduction have been reported in previous positive selection analysis, and are also observed in the present study. For instance, *PTPRC* encodes Protein tyrosine phosphatase, receptor type C, which is a member of protein tyrosine phosphatase family, and is specifically expressed in hematopoietic cells. This gene regulates T and B cell signaling and is known to be under positive selection^9^. Another example is *TSPAN8*, which encodes tetraspanin-8, a trans-membrane protein that mediates cell growth, development and is also involved in cancer^6^.

In addition to the known selected genes, we also identified novel genes with notable functions. *TTN, RYR2* and *CACNA1G,* which were among the top genes in our list, play essential roles in muscle contraction process. *TTN* encodes a large abundant protein in striated muscle, while *RYR2* and *CACNA1G* play an essential role in calcium ion transportation and muscle contraction. Another calcium channel family member in this category, *CACNA1C,* also showed a strong signal of positive selection in our analysis.

To be noted, we identified a set of genes associated with neurogenesis or neuron recognition. The most important gene in it is *EFNB3*, a member of ephrin gene family. It plays pivotal role in brain development and maintenance, particularly in forebrain function^20^. Other genes like *CELSR3* and *SLIT2*, which are involved in neural development and brain function^21^,^22^, are also fast evolving. Of note, *CACNA1C* which encodes cellular machinery regulating the flow of calcium into neurons, also plays pivotal role in neuroncognition. It affects brain circuitry involved in thinking, attention, emotion and memory. Recent evidence has demonstrated its association with mental illnesses, including schizophrenia and bipolar disorder^23^. The early adaption of this gene may have been involved in the development of primate intelligence.

## Discussion

We have introduced a powerful, efficient and accurate tool to study the genetics of NHP, in particular the macaque, which has become the essential pre-clinical animal model for drug discovery, vaccine development and biomedical research. Exome sequencing could be adopted to uncover most functional genomic variations in protein coding regions, and thus provide relevant genotypic information to explain disease, physiology and developmental regulation. Compared with whole genome assembly and resequencing, exome sequencing of NHP facilitates investigating the population genetics in large scale. Recently, human exome sequencing products have been applied to capture and sequence the NHP exome, including macaque and chimpanzee, in which positive selection was studied as proof of concept. To address their limitations, we provide the new approach, which surpasses the old one in exome coverage and efficiency. With same amount of sequencing data, our new methodology ensures more complete gene coverage, greater sequencing depth, and calling more variations accurately, and therefore provides cost effectiveness. The new probes for target enrichment were designed directly on macaque genome, which not only ensure the inclusion of more divergent regions with human genome, but also exclude the possibility of missing macaque unique genes and gene duplications. Through head-to-head comparison of samples and sample replicates, substantial genes/exons, including biomedical targets, were more efficiently captured in the new approach. Functional annotation revealed these genes played essential roles in various cellular processes.

In this study we performed a comprehensive and powerful experiment to test and validate the improved efficiency of the new approach. We tested four species/subspecies of macaque with two animals each, and ran four technique replications to detect the systematic differences instead of the stochastic process. Our result clearly illustrate the platform effect on gene coverage and mutation calling. Additionally, we processed the same samples concurrently on both the old (HP) and new (MP) platform, thus allowing head-to-head comparison between the two. The adequate sample size and representative sample selection to test the method ensured the reliability and confidence of this study, as well as the performance of the new approach. Our results have important implications on applications for the field. Eric J Valender in his HP approach demonstrated that, within rhesus exome, gene coverage was correlated with sequence identity/divergence to human, which dropped fairly rapidly when the divergence was greater than 5%^10^. Conceivably, this resulted from the lower kinetics of hybridization reaction with the greater nucleotide mismatches between rhesus coding sequences and the probes designed from human reference. Our result, on the other hand, has directly demonstrated that filling this gap results in enhanced coverage on those divergent genes. In this perspective, we could envision that MP probes would work better than HP in other NHP species within the branch of old world monkey, including baboon (*Papio* sp.), velvet monkey (*Cholorocebus aethiops*) and colobus monkey (*Colobus angolensis*), as the divergence of coding sequences between the old world monkeys should be lower than their deviation from human. Our attempt suggests that, with the substantial efforts to sequence and assemble the genome of NHPs in different branches of phylogeny, species specific probes are beneficial to capturing and uncovering the diversity of coding genome for NHP. The new method to capture and sequence other species of NHP should be approached cautiously, with preparation of several representative sets of probes within the phylogeny being optimal. This recommendation would also extend to the practice of sequencing genetically related species by probes from other representative genomes, in regions other than exome.

Utilizing the data generated by the new approach, we have attempted to provide some valuable analysis on evolutionary genetics. By characterizing the mutations containing stop codons and frameshift indels, we have identified candidate genes intact in human but pseudogenized in macaque. But it should be noted that the results need to be interpreted cautiously, since they might arise from imperfect NHP genome assembly or annotation, rather than a real pseudogenization event. Another powerful analysis is the scanning of positively selected genes in primate evolutionary history. Compared with previous research conducting similar analysis, our data provide more complete gene sequences^9^,^11^, which results in a more accurate and complete search on positively selected genes, as selection on partial gene may not represent the situation in whole. Finally, we uncovered abundant novel genes under positive selection across all branches of primates that have not been identified before, including the genes with function related to muscle contraction and neurogenesis.

## Material and Methods

### Samples and DNA extraction

Peripheral blood was collected from Chinese rhesus, Vietnamese cynomolgus, Indonesian cynomolgus and Mauritian cynomolgus, with two adult individuals per sub-species. The Chinese rhesus and Vietnamese cynomolgus macaque individuals were inhabited in Guangzhou, China, in which one Vietnamese cynomolgus individual was whole-genome short-reads sequenced and assembled in the paper of 2011^13^. The Indonesian cynomolgus individuals were inhabited in Bogor, Indonesian and the Mauritian cynomolgus animals were from Senneville, Mauritius. The origins of these individuals were confirmed by mitochondrial DNA sequencing. Genomic DNA was extracted from the peripheral blood with commercial kits. All the experiments on Chinese rhesus and Vietnamese cynomolgus animals involved in this study have been approved by the institutional committee in South China University of Science and Technology. The Indonesian and Mauritian cynomolgus monkeys samples were sourced from Maccine Pte Ltd. Approvals from Maccine’s Institutional Animal Care and Use Committee (IACUC) as well as from Merck IACUC were obtained to use any sample. We adhered to the guidelines for the care and use of animals for scientific purposes established by the Singapore National Advisory Committee for Laboratory Animal Research (NACLAR) in November 2004.

### Exome sequences extraction and probes design

To design a chip which can capture macaque exome in the genus *Macaca* efficiently, we extracted the coordinates from 187619 Consensus Coding Sequences (CCDS) of CE and 189751 CCDS of CR as annotated in the paper of 2011^13^. In order to remove the redundancy, we used the result from the pairwise-blastz alignment of CDS sequences of CE and CR, and then merged the orthologous regions which we defined as less 3 bp mismatches existed between. To improve the efficiency of probes to capture the 5′ and 3′ end of coding sequences, a 100 bp flanking sequence was added to both sides of any target sequences after merging. Finally, we obtained 52.1 Mb final target regions (fTRs), covering 35.9 Mb exonic regions. The fTRs were sent to Nimblegen (Roche Nimblegen Inc.) to design targeted probes according to the manufacturer’s pipeline.

### Library construction and exome sequencing

HP libraries were prepared and exome enriched in the Agilent SureSelect Human all exon target enrichment system (Agilent Technologies) following manufacturer’s instructions. Briefly, genomic DNA was fragmented to 200 bp by Covaris S2 (Covaris Inc.), and then was end repaired, A-tailed and adaptor ligated. The product was purified with Ampure beads and amplified with 6 cycles’ ligation mediated PCR. Exome enrichment was conducted by Agilent’s pipelines and the enriched product was further amplified by PCR to be prepared for sequencing. MP libraries were prepared and exome captured following Nimblegen sequence capture protocols. All the libraries were then subjected to quantification, cluster generation and 100 bp pair-end sequencing in Hiseq 2000 platform (Illumina Inc.) following the manufacturer’s instructions.

### Alignment and variation calling

After raw data process to generate clean data, SOAPaligner/SOAP2 version 2.20 was adopted to align the short reads to the reference genome by the parameters of -a –b –u -2 -D -v 5 -l 35 -s 40 -m 0 -x 500 -p 4 -r 1 -n 0. IR assembly with GenBank accession of GCA_000002255.2, CE assembly of GCA_000230815.1 and CR assembly of GCA_000230795.1 were used as alignment reference genomes. After alignment, the basic statistics in Table S1 were calculated and summarized. SNPs were called by the SOAPsnp in the SOAP2 version 2.20 package. To call indels accurately, we used BWA to align the reads to reference genome (aln -o 1 -e 63 -i 15 -IL -l 31 -k 2 -t 6) and used SAMtools with default parameters to call indels, and further filtered with Q20 and depth = 4 to get the final result.

### Biomedical genes analysis

We downloaded the gene list from Drugbank database^14^, in which 2171 human genes were consisted in the category of Target links, 110 genes were consisted in the category of Transporter links, 171 were contained in the list of Enzyme links and 11 were in the Carrier links (details not shown). Their sequences were BLAT to the CE and CR reference genome and 2146 genes were found to have orthologs (list in Table S4). To analyze and compare the sequence coverage of these genes in both HP and MP platform, we used SOAP2 to align the reads of all samples to the human genome (-v 5 -l 35 -s 40 -m 0 -x 500 -p 4 -r 1 -n 0) and SoapCoverage (-cvg) was then applied to calculate the coverage, depth and other statistics for these biomedical genes.

### Gene pseudogenization analysis

To yield high confident sites mutated compared to human genome (hg19), SOAP2 and BWA were deployed to align sequences from 8 individuals onto human reference genome, then SOAPsnp and SAMtools were used to call variants. Base filtering on sequencing depth and quality was also applied to remove low quality variants. Annovar (version released on November 20, 2011) was used to annotate filtered variants to illustrate their impacts on potential protein products. For a gene, if it was found to carry a homozygous premature stop-gain SNP or a frameshift indel mutation, it was regarded that its translational protein had been dramatically disrupted and would not be able to carry its original function. We counted the number of animals that carried those stop-gain or frameshift mutations, and defined the genes carrying the mutation in all 8 individuals as pseudogenized genes.

### Experimental validation of the gene pseudogenization

We randomly selected several representative gene pseudogenization events, especially those with biomedical implications and are discussed in the result section of the paper, to do the PCR plus Sanger sequencing validation. Primers were designed according to the flanking sequences of each homozygous stop-codon gain SNV or frameshift indel to warrant the successful PCR amplification. PCR was performed on additional animals beyond the ones used in the paper, for 5 individuals per Chinese rhesus and Vietnamese cynomolgus, to ensure the reliability of the pseudogenization events. PCR products were directly sequenced on 3730x DNA analyzer (Applied Biosystems, Life technologies).

### Positive selection analysis

#### Phylogenetic relationship determination

To scan for positive genes using PAML, we firstly have to determine phylogenetic relationship of these species, to do that, coding sequences of 583 randomly chosen orthologous genes from 13 individuals were concatenated, and analyzed by PhyML^24^, a software that estimates maximum likelihood phylogenies from alignments of nucleotide or amino acid sequences, using default parameters. Species include human (HM), chimpanzee (CM), Indian rhesus macaque (IR), Chinese rhesus macaque (CR), Vietnamese cynomolgus macaque (CC), Indonesian cynomolgus macaque (IC) and Mauritian cynomolgus macaque (MC). Individuals from the same species all had consistent position in the inferred phylogenetic tree, and their phylogenetic topology was determined as ((CM,HM),(IR,(CR,(CC,(IC,MC))))) (Figure S5), which was used for downstream dN/dS calculation and positive genes detection.

#### Filtering of orthologs and sequences

Orthologs were firstly determined as described^13^. Sequences of 13 individuals from 9 species/subspecies were aligned, and we filtered for orthologs containing no non-sense, frameshift mutation in at least 6 species/subspecies, by which a total of 13503 orthologs passed. Block substitutions within coding region may come from mapping errors or sequencing problems, which could give rise to misleading positive selection signal or problematic phylogenic tree. Before orthologs were used for positive selection analysis and tree inferring, block substitutions, defined by 6 or more substitutions within a windows of 9 base pairs, were masked for further analysis.

#### Positive selection analysis

For each species/subspecies, one individual from MP experiment was selected for positive selection analysis. To test for positive selection across all branches of primate phylogeny, we compared M7 and M8 Model in Codeml program, and performed a likelihood ratio test to calculate P-values, q-value was also calculated using qvalue package in R language to estimate their FDR levels.

Codeml program in PAML assumes that the tree used to calculate the positive selection represents the true evolutionary history of the aligned sequences. Both species tree and gene tree could be appropriate for this analysis in different circumstances. To ensure the robustness and reduce false positive hits, we applied a stringent pipeline as follows. At first, we tested the positive selection under the species tree. For the 209 genes we found to be positively selected under species tree (FDR < 0.1), their gene trees were also inferred by PhyML, and were used to repeat the positive selection analysis to see if the signals still existed. Genes calculated with FDR less than 0.1 in both species tree and gene tree were retained as candidates for positively selected genes. 187 genes were able to pass this filter and included in our final list.

In the result section, all of the FDRs and P values stated were inferred from the species tree, other than indicated.

Parameters used in M7-M8 model for searching for genes under positive selection are listed as follows:

runmode = 0

seqtype = 1

CodonFreq = 3

aaDist = 0

aaRatefile = wag.dat

model = 0

NSsites = 7 8

icode = 0

Mgene = 0

fix_kappa = 0

kappa = 2

fix_omega = 0

omega = 1.0

fix_alpha = 1

alpha = 0

Malpha = 0

ncatG = 10

clock = 0

getSE = 0

RateAncestor = 1

cleandata = 0

ndata = 1

method = 0

## Additional Information

**Accession codes**: Sequence data from this article have been deposited with the NCBI Sequence Read Archive (SRA) under Accession No. SRA261275.

**How to cite this article**: Su, Z. *et al*. Species specific exome probes reveal new insights in positively selected genes in nonhuman primates. *Sci. Rep.* **6**, 33876; doi: 10.1038/srep33876 (2016).

## Supplementary Material

Supplementary Information

Supplementary Information

## Figures and Tables

**Table 1:** Samples, replicates and aligning references used in this study.

Sample ID	Population	Capture array	No. of replicates	Reference
MC1-HP	Mauritian cynomolgus macaque	HP	4	IR/CE
MC2-HP	Mauritian cynomolgus macaque	HP	4	IR/CE
IC1-HP	Indonesian cynomolgus macaque	HP	4	IR/CE
IC2-HP	Indonesian cynomolgus macaque	HP	4	IR/CE
MC1-MP	Mauritian cynomolgus macaque	MP	4	IR/CE
MC2-MP	Mauritian cynomolgus macaque	MP	4	IR/CE
IC1-MP	Indonesian cynomolgus macaque	MP	4	IR/CE
IC2-MP	Indonesian cynomolgus macaque	MP	4	IR/CE
CC1-MP	Vietnamese cynomolgus macaque	MP	1	IR/CE
CC2-MP	Vietnamese cynomolgus macaque	MP	1	IR/CE
CR1-MP	Chinese rhesus macaque	MP	1	IR/CR
CR2-MP	Chinese rhesus macaque	MP	1	IR/CR

**Table 2:** The top 50 genes showing evidence for positive selection.

**Gene name**	**Ensembl protein ID**	**Chr**	**description**	P value*	FDR*
*TTN*	ENSP00000348444	2	titin	8.29E-41	7.15E-37
*RYR2*	ENSP00000355533	1	ryanodine receptor 2 (cardiac)	6.04E-26	3.47E-22
*C9orf93*	ENSP00000370077	9	chromosome 9 open reading frame 93	1.69E-13	7.27E-10
*PRDM9*	ENSP00000296682	5	PR domain containing 9	5.78E-12	1.66E-08
*ATF7IP2*	ENSP00000348799	16	activating transcription factor 7 interacting protein 2	1.87E-11	4.14E-08
*CELSR2*	ENSP00000271332	1	cadherin, EGF LAG seven-pass G-type receptor 2	1.92E-11	4.14E-08
*SYDE1*	ENSP00000341489	19	synapse defective 1, Rho GTPase, homolog 1 (C. elegans)	8.22E-11	1.58E-07
*ANO8*	ENSP00000159087	19	anoctamin 8	1.02E-10	1.76E-07
*PCNT*	ENSP00000352572	21	pericentrin	1.43E-10	2.24E-07
*SF1*	ENSP00000366604	11	splicing factor 1	2.11E-10	3.04E-07
*SLIT2*	ENSP00000273739	4	slit homolog 2 (Drosophila)	2.46E-10	3.27E-07
*PITPNM2*	ENSP00000322218	12	phosphatidylinositol transfer protein, membrane-associated 2	1.13E-09	1.39E-06
*APC2*	ENSP00000233607	19	adenomatosis polyposis coli 2	2.43E-09	2.79E-06
*DENND4B*	ENSP00000354597	1	DENN/MADD domain containing 4B	3.01E-09	3.04E-06
*DZIP3*	ENSP00000419981	3	DAZ interacting protein 3, zinc finger	3.08E-09	3.04E-06
*PTPRC*	ENSP00000271610	1	protein tyrosine phosphatase, receptor type, C	3.18E-09	3.04E-06
*TACC2*	ENSP00000358001	10	transforming, acidic coiled-coil containing protein 2	9.29E-09	8.44E-06
*C2orf16*	ENSP00000386190	2	chromosome 2 open reading frame 16	1.09E-08	9.36E-06
*CACNA1G*	ENSP00000352011	17	calcium channel, voltage-dependent, T type, alpha 1G subunit	1.42E-08	1.15E-05
*CCDC144A*	ENSP00000353717	17	coiled-coil domain containing 144A	2.17E-08	1.62E-05
*COL4A3*	ENSP00000379823	2	collagen, type IV, alpha 3 (Goodpasture antigen)	3.53E-08	2.53E-05
*SALL1*	ENSP00000251020	16	sal-like 1 (Drosophila)	3.85E-08	2.55E-05
*KIF1A*	ENSP00000362405	2	kinesin family member 1A	4.08E-08	2.61E-05
*PIBF1*	ENSP00000317144	13	progesterone immunomodulatory binding factor 1	4.88E-08	2.92E-05
*QRICH2*	ENSP00000262765	17	glutamine rich 2	5.38E-08	3.09E-05
*MAPK14*	ENSP00000229794	6	mitogen-activated protein kinase 14	6.32E-08	3.51E-05
*FOXP1*	ENSP00000420736	3	forkhead box P1	7.24E-08	3.90E-05
*EYA4*	ENSP00000395916	6	eyes absent homolog 4 (Drosophila)	7.91E-08	4.13E-05
*SI*	ENSP00000264382	3	sucrase-isomaltase (alpha-glucosidase)	9.48E-08	4.81E-05
*FAM111B*	ENSP00000341565	11	family with sequence similarity 111, member B	1.02E-07	5.02E-05
*FCRL3*	ENSP00000357169	1	Fc receptor-like 3	1.32E-07	6.33E-05
*CARD10*	ENSP00000384570	22	caspase recruitment domain family, member 10	1.42E-07	6.62E-05
*CLEC4F*	ENSP00000272367	2	C-type lectin domain family 4, member F	3.24E-07	1.47E-04
*IL1RAP*	ENSP00000408893	3	interleukin 1 receptor accessory protein	4.08E-07	1.80E-04
*PLXNB1*	ENSP00000351338	3	plexin B1	4.30E-07	1.85E-04
*TSPAN8*	ENSP00000377003	12	tetraspanin 8	6.07E-07	2.55E-04
*ADAMTS4*	ENSP00000356975	1	ADAM metallopeptidase with thrombospondin type 1 motif, 4	9.23E-07	3.70E-04
*DMRTA2*	ENSP00000383909	1	DMRT-like family A2	1.08E-06	4.22E-04
*CALU*	ENSP00000420381	7	calumenin	1.14E-06	4.29E-04
*SNRPB*	ENSP00000342305	20	small nuclear ribonucleoprotein polypeptides B and B1	1.14E-06	4.29E-04
*SLC26A3*	ENSP00000345873	7	solute carrier family 26, member 3	1.33E-06	4.79E-04
*MEGF6*	ENSP00000398045	1	multiple EGF-like-domains 6	1.37E-06	4.83E-04
*VAV2*	ENSP00000360916	9	vav 2 guanine nucleotide exchange factor	1.43E-06	4.94E-04
*ARID1B*	ENSP00000356116	6	AT rich interactive domain 1B (SWI1-like)	1.47E-06	4.97E-04
*CELSR3*	ENSP00000164024	3	cadherin, EGF LAG seven-pass G-type receptor 3 (flamingo homolog, Drosophila)	1.84E-06	6.09E-04
*TYK2*	ENSP00000264818	19	tyrosine kinase 2	2.36E-06	7.55E-04
*EAF2*	ENSP00000410708	3	ELL associated factor 2	2.47E-06	7.74E-04
*CLCA4*	ENSP00000263723	1	chloride channel accessory 4	2.66E-06	8.18E-04
*MTCL1*	ENSP00000396032	18	microtubule crosslinking factor 1	3.55E-06	1.06E-03
*SIK3*	ENSP00000391295	11	SIK family kinase 3	3.56E-06	1.06E-03

^*^Genes were ranked by P values. The P value and FDR were inferred from the species tree. Refer to Table S8 for the gene-tree result.

## References

[b1] Abee C. R. Nonhuman primates in biomedical research Second edition (Academic Press, 2012).

[b2] Chen Y., Niu Y. & Ji W. Transgenic nonhuman primate models for human diseases: approaches and contributing factors. Journal of genetics and genomics = Yi chuan xue bao 39, 247–251, doi: 10.1016/j.jgg.2012.04.007 (2012). 22749011

[b3] T Hart B. A., Bogers W. M., Haanstra K. G., Verreck F. A. & Kocken C. H. The translational value of non-human primates in preclinical research on infection and immunopathology. European journal of pharmacology 759, 69–83, doi: 10.1016/j.ejphar.2015.03.023 (2015). 25814254

[b4] Verdier J. M. . Lessons from the analysis of nonhuman primates for understanding human aging and neurodegenerative diseases. Frontiers in neuroscience 9, 64, doi: 10.3389/fnins.2015.00064 (2015). 25788873 PMC4349082

[b5] Phillips K. A. . Why primate models matter. American journal of primatology 76, 801–827, doi: 10.1002/ajp.22281 (2014). 24723482 PMC4145602

[b6] Gibbs R. A. . Evolutionary and biomedical insights from the rhesus macaque genome. Science 316, 222–234, doi: 10.1126/science.1139247 (2007). 17431167

[b7] Hodges E. . Genome-wide *in situ* exon capture for selective resequencing. Nature genetics 39, 1522–1527, doi: 10.1038/ng.2007.42 (2007). 17982454

[b8] Albert T. J. . Direct selection of human genomic loci by microarray hybridization. Nature methods 4, 903–905, doi: 10.1038/nmeth1111 (2007). 17934467

[b9] George R. D. . Trans genomic capture and sequencing of primate exomes reveals new targets of positive selection. Genome research 21, 1686–1694, doi: 10.1101/gr.121327.111 (2011). 21795384 PMC3202285

[b10] Vallender E. J. Expanding whole exome resequencing into non-human primates. Genome biology 12, R87, doi: 10.1186/gb-2011-12–9-r87 (2011). 21917143 PMC3308050

[b11] Hvilsom C. . Extensive X-linked adaptive evolution in central chimpanzees. Proceedings of the National Academy of Sciences of the United States of America 109, 2054–2059, doi: 10.1073/pnas.1106877109 (2012). 22308321 PMC3277544

[b12] Jin X. H. M., Ferguson B., Meng Y., Ouyang L. . An Effort to Use Human-Based Exome Capture Methods to Analyze Chimpanzee and Macaque Exomes. Plos One 7, 10, doi: 10.1371/journal.pone.0040637 (2012). PMC3407233 22848389

[b13] Yan G. . Genome sequencing and comparison of two nonhuman primate animal models, the cynomolgus and Chinese rhesus macaques. Nature biotechnology 29, 1019–1023, doi: 10.1038/nbt.1992 (2011). 22002653

[b14] Wishart D. S. . DrugBank: a knowledgebase for drugs, drug actions and drug targets. Nucleic acids research 36, D901–D906, doi: 10.1093/nar/gkm958 (2008). 18048412 PMC2238889

[b15] Smith R. L., Warren R. B., Griffiths C. E. & Worthington J. Genetic susceptibility to psoriasis: an emerging picture. Genome medicine 1, 72, doi: 10.1186/gm72 (2009). 19638187 PMC2717398

[b16] Allanore Y. . Genome-wide scan identifies TNIP1, PSORS1C1, and RHOB as novel risk loci for systemic sclerosis. PLoS genetics 7, e1002091, doi: 10.1371/journal.pgen.1002091 (2011). 21750679 PMC3131285

[b17] Wilming L. G. . Sequencing and comparative analysis of the gorilla MHC genomic sequence. Database: the journal of biological databases and curation 2013, bat011, 10.1093/database/bat011 (2013). 23589541 PMC3626023

[b18] Yang Z. PAML 4: phylogenetic analysis by maximum likelihood. Molecular biology and evolution 24, 1586–1591, doi: 10.1093/molbev/msm088 (2007). 17483113

[b19] Nielsen R. . A scan for positively selected genes in the genomes of humans and chimpanzees. PLoS biology 3, e170, doi: 10.1371/journal.pbio.0030170 (2005). 15869325 PMC1088278

[b20] Hruska M. & Dalva M. B. Ephrin regulation of synapse formation, function and plasticity. Molecular and cellular neurosciences 50, 35–44, doi: 10.1016/j.mcn.2012.03.004 (2012). 22449939 PMC3631567

[b21] Tissir F. . Lack of cadherins Celsr2 and Celsr3 impairs ependymal ciliogenesis, leading to fatal hydrocephalus. Nature neuroscience 13, 700–707, doi: 10.1038/nn.2555 (2010). 20473291

[b22] Itoh A., Miyabayashi T., Ohno M. & Sakano S. Cloning and expressions of three mammalian homologues of Drosophila slit suggest possible roles for Slit in the formation and maintenance of the nervous system. Brain research. Molecular brain research 62, 175–186 (1998). 9813312 10.1016/s0169-328x(98)00224-1

[b23] Identification of risk loci with shared effects on five major psychiatric disorders: a genome-wide analysis. Lancet 381, 1371–1379, doi: 10.1016/S0140-6736(12)62129-1 (2013). 23453885 PMC3714010

[b24] Guindon S. . New algorithms and methods to estimate maximum-likelihood phylogenies: assessing the performance of PhyML 3.0. Systematic biology 59, 307–321, doi: 10.1093/sysbio/syq010 (2010). 20525638

