# Supplementary material for: Species specific exome probes reveal new insights in positively selected genes in nonhuman primates
Source: Sci Rep. 2016 Sep 23;6:33876. doi: 10.1038/srep33876 (PMC5034232; doi:10.1038/srep33876)
Supplement: Supplementary Information [file srep33876-s2.pdf]

# Supplementary Information for

# Species specific exome probes reveal new insights in positively selected genes in nonhuman primates

Zheng Su[1,a], Junjie Zhang[2,a], Chanchal Kumar[3,a,b], Cliona Molony[4], Hongchao Lu[5], Ronghua Chen[5,6], David J. Stone[7], Fei Ling[2], Xiao Liu[1, 8]

[1]BGI-Shenzhen, Shenzhen, Guangdong 518083, China;
[2]Shool of bioscience & bioengineering, South China University of Technology, Guangzhou, Guangdong 510006, China;
[3]Translational Medicine Research Centre, Merck Research Laboratories, MSD, 8 Biomedical Grove, Neuros #04-01, Singapore 138665, Singapore;
[4]Merck Research Laboratories, Merck & Co. Inc., 33 Avenue Louis Pasteur, Boston, MA 02115, USA;
[5]Informatics IT, MSD R&D (China) Co., Ltd., Beijing, China ;
[6]Informatics IT, Merck & Co., Inc., Boston, MA, USA;
[7]Merck Research Laboratories, Merck & Co. Inc., 770 Sumneytown Pike, WP53B-120 West Point, PA 19486, USA;
[8]Department of Biology, University of Copenhagen, Copenhagen 2200, Denmark

[a] These authors contribute equally to this work.
[b]Current affiliations: Cardiovascular and Metabolic Diseases Innovative Medicines, AstraZeneca R&D, Mölndal SE-431 83, Sweden. Integrated Cardio Metabolic Centre(ICMC) , Karolinska Institutet, Novum, Blickagången 6, SE-141 57 Huddinge, Sweden

Correspondence should be addressed to X.L.(liuxiao@genomics.cn) or F.L.(fling@scut.edu.cn)

---

1 These authors contribute equally to this work.

Sequence data from this article have been deposited with the NCBI Sequence Read Archive (SRA) under Accession No **SRA261275**

**Abbreviations**: Chinese rhesus macaque (Macaca mulatta lasiota, CR); cynomolgus macaque (Macaca fascicularis) of Vietnam origin (CC); Indonesian cynomolgus macaque (IC); Mauritian cynomolgus macaque (MC); Indian rhesus (IR); Cynomolgus (CE); monkey probes(MP); human probes(HP).

Figure S1: Pairwise correlation of mRNA coverage between replicates.
Left: HP pipeline; Right: MP pipeline.

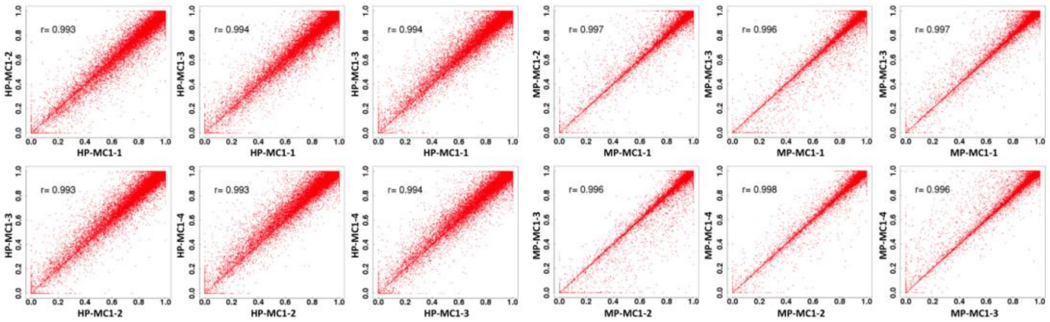


**Figure S1. Correlation of mRNA coverage between replicates of MC1.** (Left: HP; right: MP)

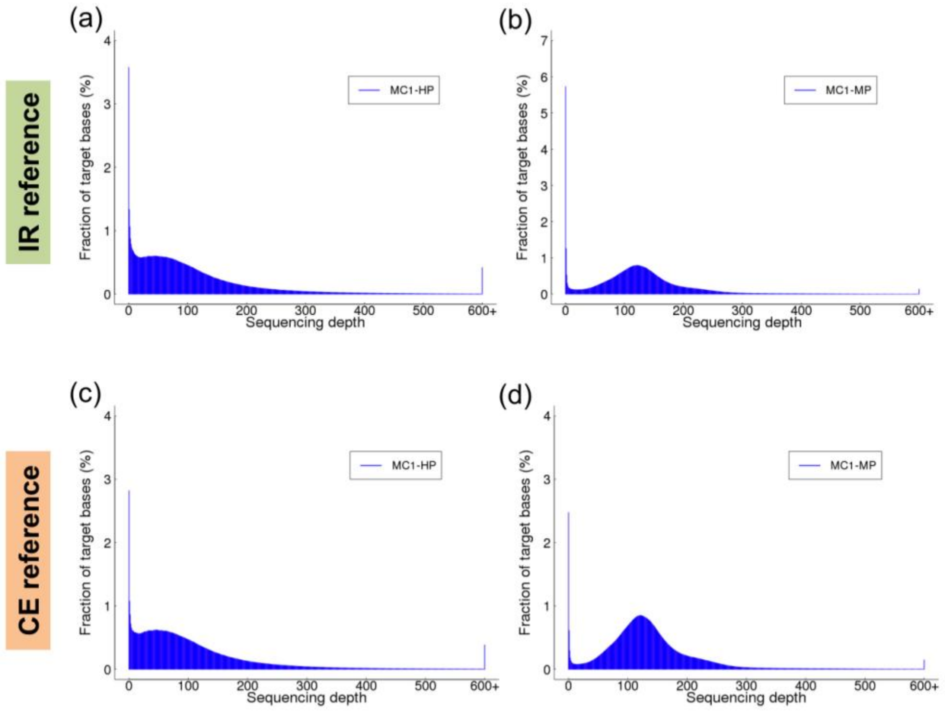


**Figure S2. The single nucleotide depth distribution of exome data of MC1 in MP and HP** (Top two figures: IR genome reference; down two figures: CE genome reference)

(a)

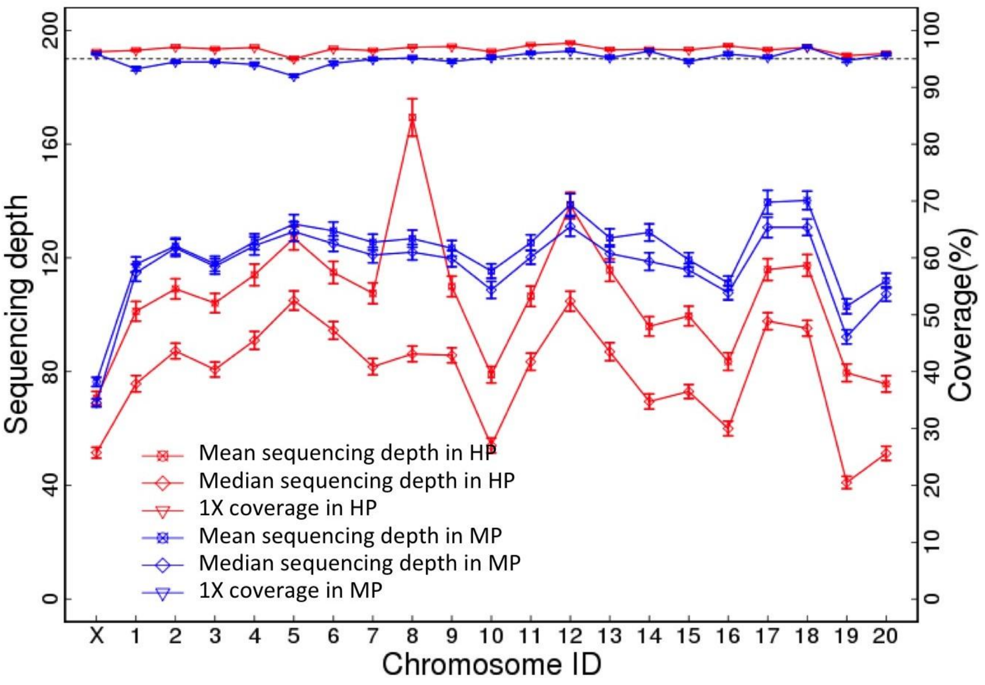


IR reference

(b)

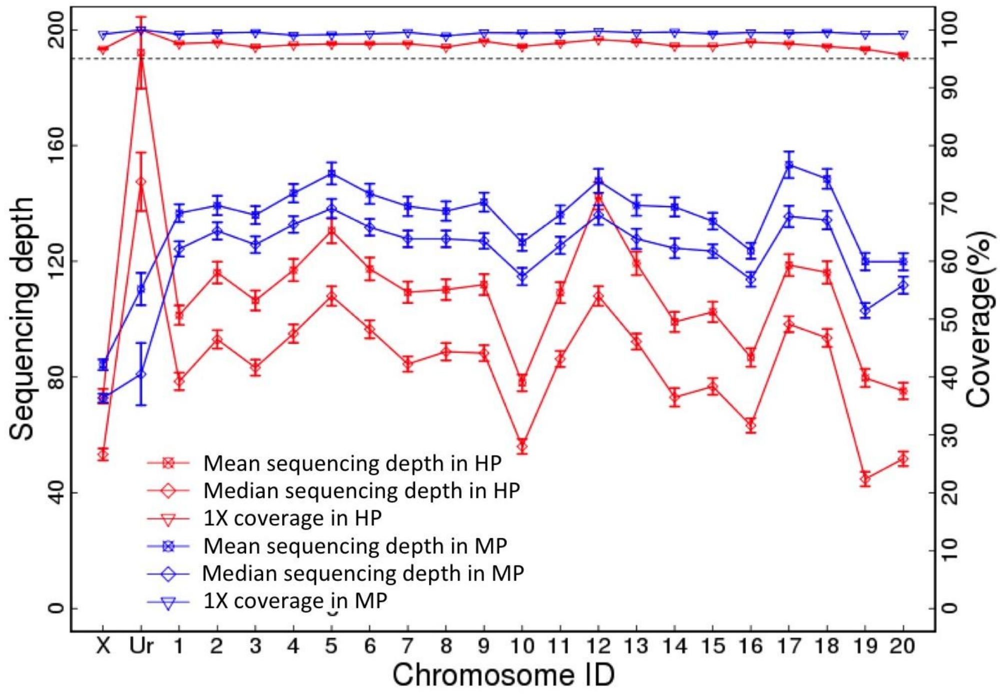


CE reference

**Figure S3. Sequence coverage, mean and median depth across all chromosomes of MC1 in HP and MP** (a: IR reference; b: CE reference)

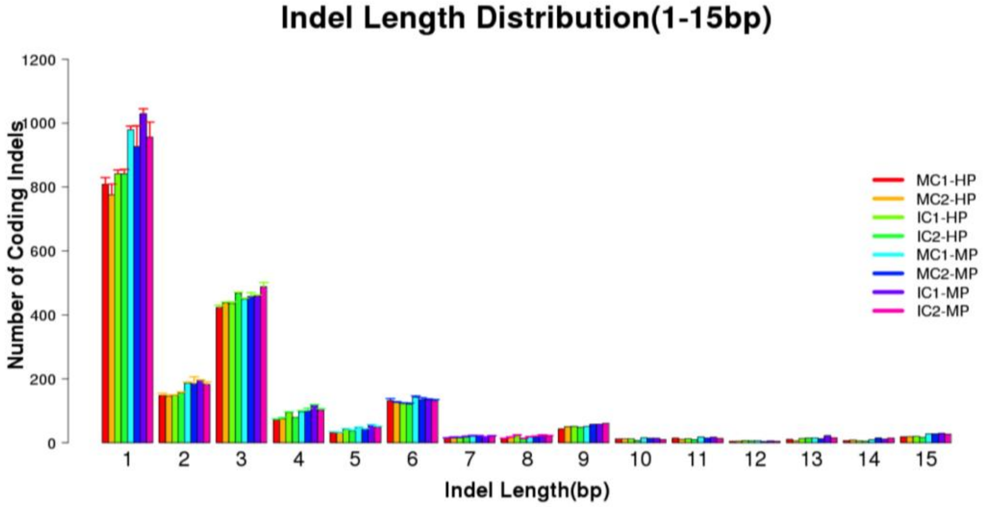


**Figure S4. The length distribution of short coding indels**

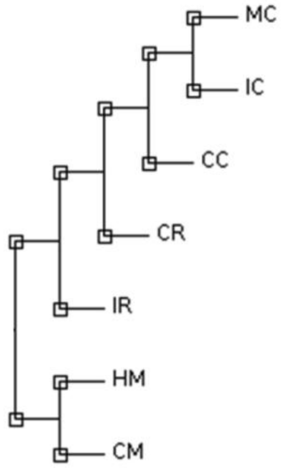


**Figure S5. The phylogenetic tree of species used in the positive selection analysis inferred by PhyML**

**HM (human), CM (chimpanzee), IR (Indian rhesus macaque), CR (Chinese rhesus macaque), CC (Vietnamese cynomolgus macaque), IN (Indonesian cynomolgus macaque), MA (Mauritian cynomolgus macaque)*
